# Extramedullary Intradural Primary Spinal Angiosarcoma: A Case Study

**DOI:** 10.7759/cureus.74767

**Published:** 2024-11-29

**Authors:** Miguel Catalo, Mariana Afonso, António Verdelho, Artur Aguiar, Mavilde Arantes

**Affiliations:** 1 Radiation Oncology, Instituto Português de Oncologia do Porto Francisco Gentil, Porto, PRT; 2 Pathology, Instituto Português de Oncologia do Porto Francisco Gentil, Porto, PRT; 3 Neurosurgery, Instituto Português de Oncologia do Porto Francisco Gentil, Porto, PRT; 4 Neuroradiology, Instituto Português de Oncologia do Porto Francisco Gentil, Porto, PRT

**Keywords:** neuro radiology, neurosurgery, primary angiosarcoma, primary intradural extramedullary spinal tumor, radiation therapy

## Abstract

Angiosarcoma is a rare soft tissue sarcoma, namely when it presents as a primary intradural extramedullary spinal neoplasm, with only one case of non-vertebral origin reported in the literature. We present the case of a 51-year-old woman with neurological symptoms of paraparesis and constipation who underwent a magnetic resonance imaging (MRI) that revealed a well-demarcated, predominantly homogeneous, intensely enhancing intradural extramedullary lesion in T2-weighted images. Histology, immunohistochemistry, and genetics of the lesion showed an angiosarcoma. The patient received adjuvant radiation therapy (RT) and is currently stable, showing no signs of recurrence, in the third year of follow-up. Our work demonstrates a rare case of extramedullary intradural primary spinal angiosarcoma, highlighting the challenges associated with its diagnosis. The multidisciplinary approach, including surgery and adjuvant RT, has been shown to effectively control the disease. Although the prognosis is generally poor, the presented case shows apparent success.

## Introduction

Angiosarcoma accounts for about 1% of all soft tissue sarcomas, which, in themselves, comprise a rare neoplasm. These originate from lymphatic or vascular endothelial cells and are categorized as aggressive due to their high grade. It is usually a spontaneous tumor; however, it can rarely arise from a benign vascular lesion [1,2]. The most frequent location is in the skin, head, neck, and scalp, although it can appear anywhere in the body, namely in the main blood vessels and heart. Extramedullary intradural spinal localization is very rare, with only five cases described [3-7], one of which is of non-vertebral origin [3].

As the tumor grows, it bleeds easily and infiltrates nearby tissues. It is often a multifocal disease that metastasizes hematogeneously to the lungs, bone, brain, lymph nodes, and other soft tissues [2,8]. It is a highly hemorrhagic lesion with a risk of rebleeding and recurrence [9].

Its frequency is similar in both genders, although it is more prevalent in older men. A connection might exist with previous irradiation, which tends to occur about a decade after RT. The mutation of BRCA1 and BRCA2 genes can also be linked with a higher probability of developing this type of tumor. Family history is highly uncommon. Chemicals such as vinyl chloride, thorium dioxide, arsenic, radium, and anabolic steroids are also associated with a higher risk [1,2]. In addition to being based on anamnesis and physical examination, the diagnosis requires adequate imaging methods to define the tumor anatomy and stage.

Finally, a biopsy of the lesion is necessary for the final diagnosis [2]. It is challenging to histologically characterize it since the morphological differences between a benign proliferation of vessels are tenuous. Microscopically, endothelial cells can be pleomorphic, rounded, polygonal, spindle-shaped, or epithelioid. When well differentiated, they form functional sinusoids. As they become less differentiated, the cells accumulate and form papilla-like projections in the vascular lumen until forming areas of hemorrhage and necrosis when completely undifferentiated. Immunohistochemistry plays a crucial role in diagnosis. Endothelial markers include endothelial factor Von Willebrand, cluster of differentiation 34 (CD34), CD31, agglutinin 1 of Ulex europaeus, and vascular endothelial growth factor (VEGF) [2,10]. According to the 2020 World Health Organization classification of soft tissue and bone neoplasms, CD31 and erythroblast transformation-specific related genes (ERG) are highly specific and sensitive in diagnosing angiosarcoma [9].

Treatment of angiosarcoma depends on its stage at presentation. In non-metastatic disease, resection with wide negative microscopic margins is the treatment of choice and is associated with better overall survival. Microscopic or macroscopic residual tumor resections confer a worse prognosis [2,8]. Being a high-grade malignant disease, perioperative RT is recommended, associated with increased survival and greater local control [8]. The use of neoadjuvant chemotherapy may be considered in large tumors where excision with disease-free margins is difficult to achieve [1]. In patients with locally advanced cutaneous angiosarcoma, the weekly addition of chemotherapy to RT has been demonstrated to improve local control rates and prolong survival [11]. In the case of localized visceral angiosarcoma, a multimodal approach with surgery, RT, and chemotherapy is recommended. In metastatic disease, chemotherapy is the treatment of choice. The first line of treatment includes paclitaxel or anthracyclines, such as doxorubicin, with response rates comparable to those of the former [11]. There is not much evidence of chemotherapy in treating central nervous system angiosarcoma [9].

Globally, it has an overall survival (OS) of about 35% at five years. When resected with microscopically negative margins, localized disease is associated with a 60% OS at five years. Advanced age, metastatic disease at diagnosis, and poor Performance Status are bad predictors of response [2,12,13].

This clinical case illustrates a rare example of extramedullary intradural primary spinal angiosarcoma, highlighting the challenges associated with its diagnosis and treatment, although with apparent success.

## Case presentation

A 51-year-old woman with no history of relevant familial neoplasms presented at the consultation in March 2021 with severe low back pain, not controlled with paracetamol, codeine, and tapentadol, progressive paraparesis, and constipation. There were no changes in bladder function. Past history was unremarkable, with only a history of hepatic hemangiomas and benign breast nodules. The patient denied trauma or previous lumbar surgery. Physical examination showed grade four paraparesis (Medical Research Council muscle power scale) in the right lower limb and grade three in the left lower limb, allodynia on the back of the ipsilateral leg, and hypoesthesia on the left foot.

**Figure 1:**
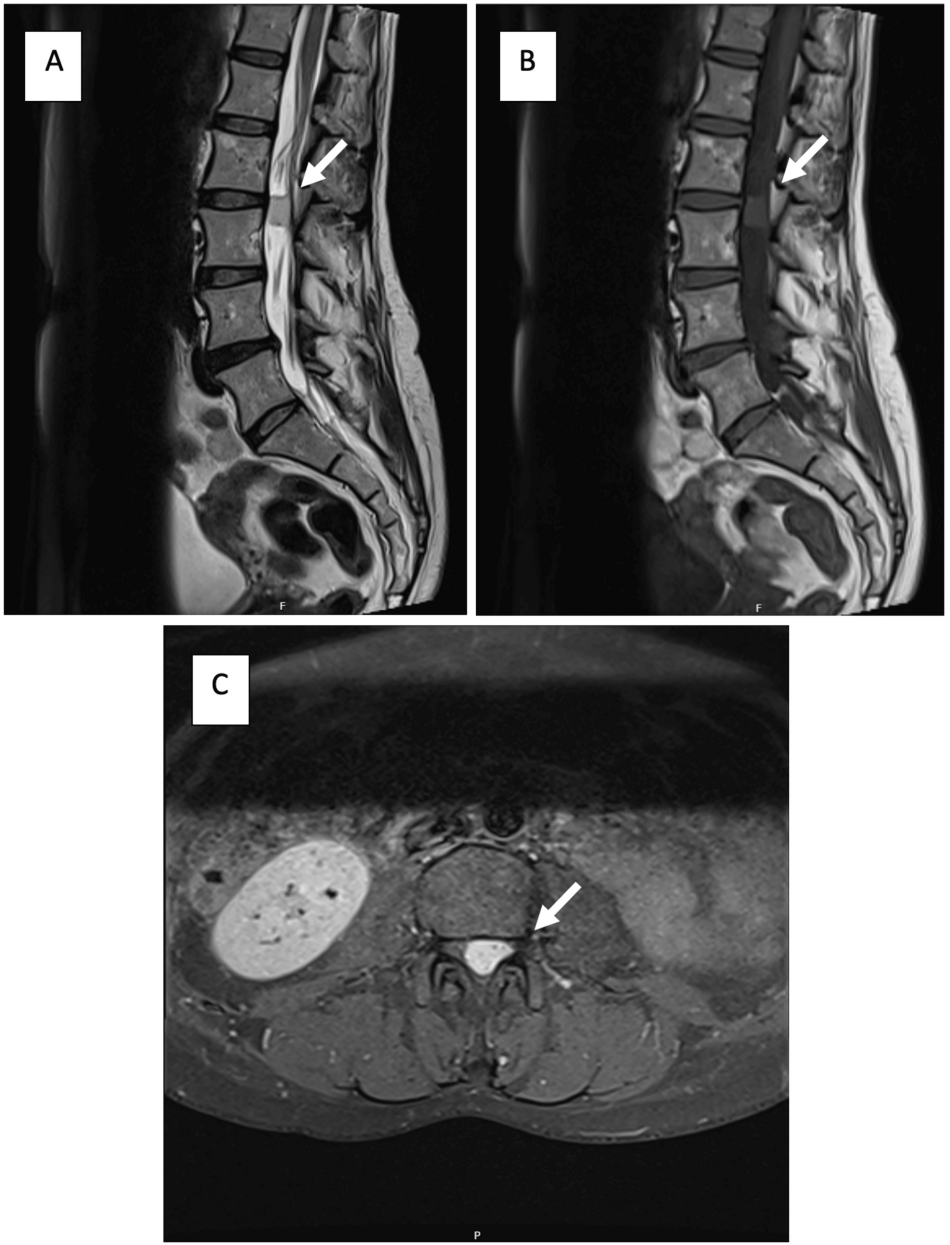
MRI showing an intracanal extramedullary lesion with compression of the cauda equina A) Sagittal T2-weighted images showed a hyperintense intradural extramedullary lesion at the level of L2-L3 with well-defined borders. B) Sagittal T1-weighted images revealed a hyperintense lesion relative to the spinal cord. C) Strong enhancement of the lesion on post-contrast axial T1-weighed imaging.

In view of these clinical findings, the patient underwent a lumbar magnetic resonance imaging (MRI) that showed a well-demarcated, predominantly homogeneous, and intensely hyperintense intracanal extramedullary lesion with cauda equina compression in T2-weighted images (Figure 1). An urgent surgical resection was performed.

Hence, in April 2021, a laminectomy and excision of the lesion was performed. An image intensifier was used to guide the incision and spinous fractures were made according to Senegás technique. The tumor was bleeding, placed anteriorly, and adherent to one of the spinal roots. Given the adherent nature of the lesion, the tumor had to be fragmented and one of the roots was sectioned with the aid of neurophysiological monitoring.

**Figure 2:**
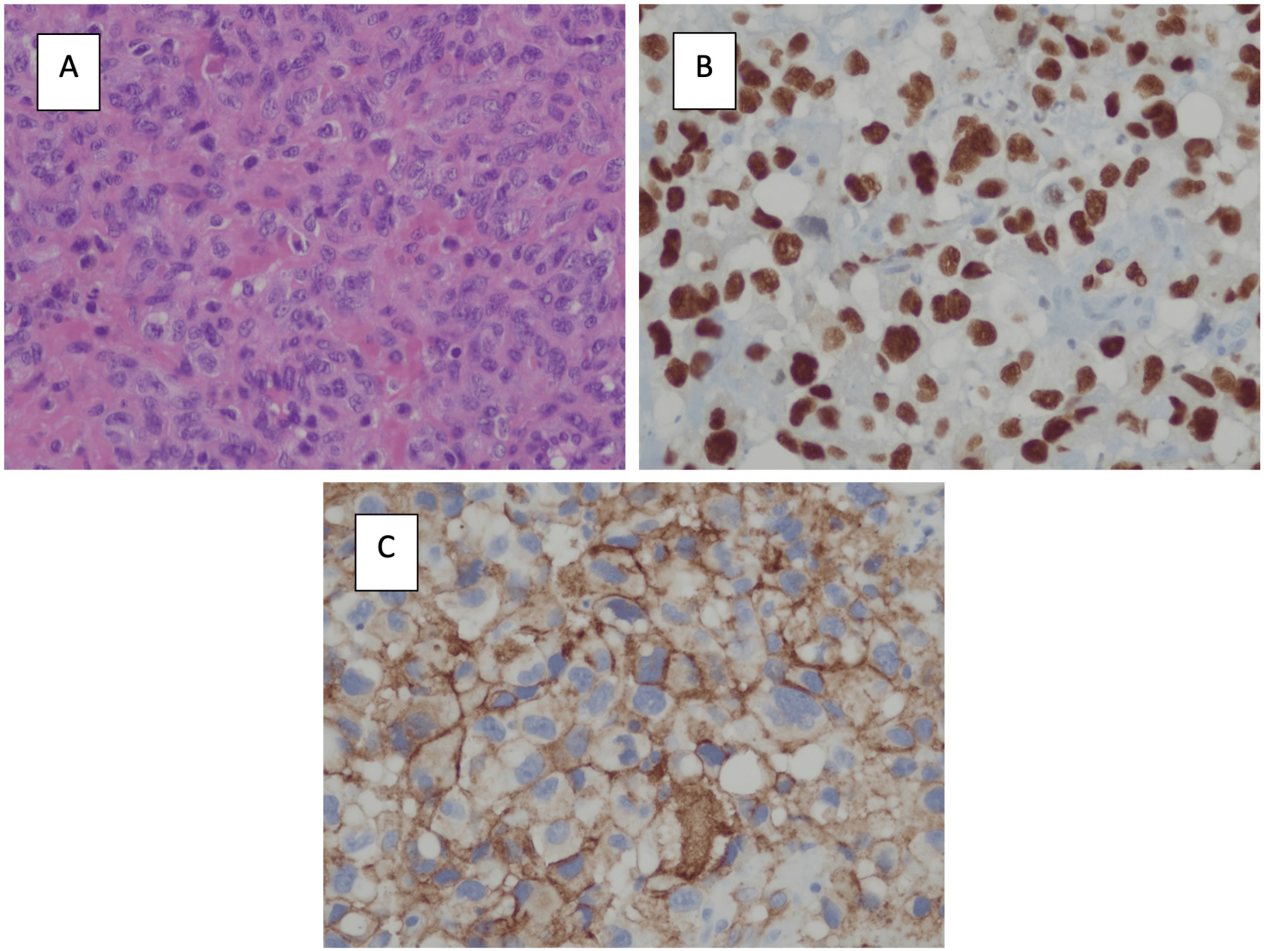
Microscope images of the resected angiosarcoma A) Hematoxylin-eosin staining of the resected angiosarcoma; B) Showing diffuse ERG staining; C) Strong endothelial expression of CD31 ERG: Erythroblast transformation-specific Related Gene

The histology of the specimen (Figure 2) revealed multiple fragments of vascular mesenchymal tumor, consisting predominantly of cells with a rounded or oval nucleus, with sparse cytoplasm, arranged in vascular canals of varying sizes. Other areas formed by cells with an enlarged nucleus, vesicular chromatin, inconspicuous nucleolus, and slightly clarified cytoplasm arranged in solid aggregates within a looser stroma and in trabeculae or isolation within blood lakes were observed. Numerous ecstatic vessels were visible, some of which were thrombosed. Nuclear pleomorphism was mild to moderate. There were six mitoses in ten high-power fields (HPF). No lymphovascular/perineural invasion was detected. Immunohistochemical study was diffusely positive for CD31, CD34, and ERG, negative for Anti-Cytokeratin 5.2 (Cam 5.2), transcription factor enhancer 3 (TFE3), protein S100 (PS100) and glial fibrillary acidic protein (GFAP). The proliferative index (Ki67) was greater than 50. Resection margins were non-assessable. In short, it was a plausible epithelioid hemangioendothelioma or angiosarcoma. Therefore, a genetic evaluation was requested for clarification, revealing a negative result for the WW domain-containing transcription regulator protein 1 - calmodulin binding transcription activator 1 (WWTR1-CAMTA1) fusion gene, validating the diagnosis of an angiosarcoma [14].

**Figure 3:**
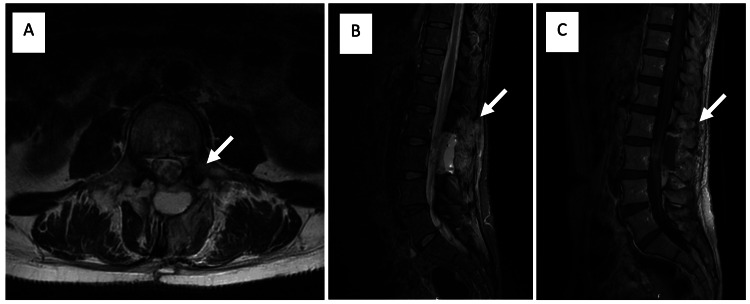
Post-operative lumbosacral spine MRI Lumbosacral MRI is showing signs of a laminectomy of L2 and L3; the presence of a left center-lateral cystic-appearing collection centered on the posterior soft tissues, measuring approximately 40 mm in cranio-caudal diameter, hyperintense on axial T2 (A) and sagittal STIR (B), hypointense on sagittal T1, without signal enhancement after gadolinium (C), probably corresponding to a seroma; and foci of signal enhancement after gadolinium in close relation with the roots of the cauda equina, possibly due to previous injury/surgical intervention (C). STIR: Sagittal short tau inversion recovery

Post-operatively, due to the previously described neurological findings, the patient began motor rehabilitation under the supervision of the physical medicine and rehabilitation team. A daily aerobic walking exercise with lower limb strength training and stretching was prescribed. Additionally, pelvic muscle floor exercises such as kegel contractions and squats were started to address constipation. This treatment was initiated during hospitalization and continued on an outpatient basis. After discharge, the patient also started hydro gymnastics, pilates, and yoga classes. One month after the intervention, the patient underwent an MRI, which showed no residual tumor (Figure 3).

**Figure 4:**
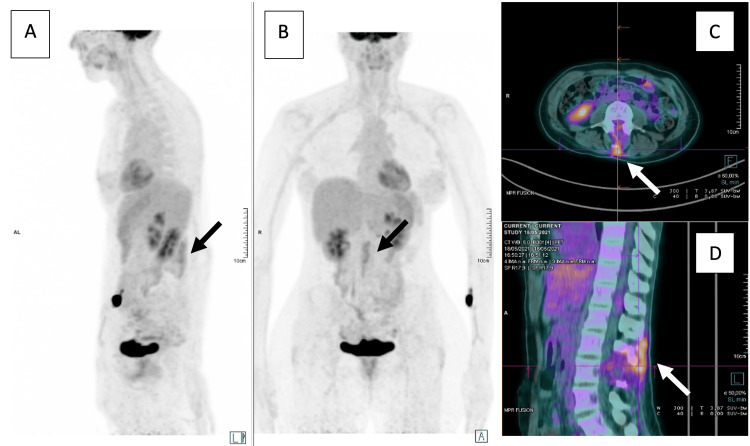
Post-operative PET/CT scan Lateral (A) and coronal (B) views of the maximal intensity projection (MIP) display, axial (C) and sagittal plane (D) of fused tomographic renditions showing high uptake in the posterior planes of the spinous apophysis of L2 and in the body of L3, probably related to iatrogenic alterations. Tumor persistence could not be excluded. No other irregular foci were observed.

In addition, a positron emission tomography (PET/CT) scan was performed, revealing uptake in the posterior planes of the spinous apophysis of L2 and the body of L3, possibly related to iatrogenic alterations or, less probably, to tumor persistence, that could not be securely excluded. The examination did not reveal any other foci (Figure 4).

Ascribable to these findings, a multidisciplinary group decided to perform adjuvant external beam radiation therapy in the intervened region. In June 2021, the patient began treatment, receiving a dose of 54 Gray (Gy) over 27 fractions, 2 Gy per fraction, once daily, five days a week, using the volumetric modulated arc therapy (VMAT) technique, with 6 MV photons targeting the surgical site (L1 to L4). With the aid of MRI images, this region and the seroma were included in the clinical target volume (CTV) with a 7 mm expansion to the planning target volume (PTV). The treatment took place with good clinical tolerance and without interruptions.

In October 2021, the patient had her first follow-up appointment for Neurosurgery. The patient presented with independent ambulation with the aid of a cane, excellent general condition, and reported being functionally autonomous in activities of daily living. Despite these improvements, the patient maintained an intense mechanical low back pain with extension to the left lower limb that had started before the end of RT. Some imbalance was also reported but without episodes of falling. Pain was adequately controlled with gabapentin and paracetamol. The patient no longer had constipation and remained free of urinary obstruction. On physical examination, there was an improvement in mono paresis of the right lower limb, allodynia on the left leg, and hypoesthesia on the ipsilateral foot. Electromyography carried out in November 2021 showed a slight bilateral lumbosacral pluriradicular compromise at the level of L3 and L4. 

**Figure 5:**
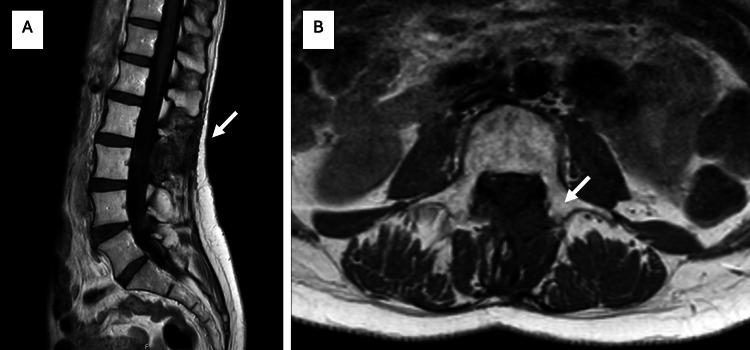
Last surveillance MRI of the spine Sagittal post-contrast T1-weighted (A) and axial T1-weighted (B) MRI showing signs of a previous laminectomy of L2 and L3. No notable foci of enhancement were visible.

On the last MRI (Figure 5) and surveillance visit in March 2024, the patient showed no disease; the pain was controlled with analgesic medication, and other alterations were in line with the previous physical examination.

## Discussion

Extramedullary primary intradural spinal angiosarcoma is a rare and challenging entity to diagnose, as evidenced by this clinical case. It has an extremely low incidence, with only one other case of non-vertebral origin described in the literature [3]. The difficulty in diagnosis and treatment lies in its rarity.

The current patient appears to have a spontaneous angiosarcoma, given the absence of a significant family or personal history, without trauma or previous surgical interventions at the site. The clinical presentation of angiosarcoma in this location may include neurological symptoms with severe low back pain and progressive paraparesis, as this patient experienced.

According to the literature, this tumor tends to appear as a well-demarcated, heterogeneous, moderately to intensely hyperintense lesion on the T2-weighted MRI sequence, with vasogenic edema [9,15]. Our lesion meets all these criteria except that it was predominantly homogeneous, probably due to its smaller size. There are possible differential diagnoses in this location, such as schwannoma, cavernous hemangioma, and metastases, all of which can present with similar imaging features.

Schwannoma presentation is similar to angiosarcoma's, showing a heterogeneous, iso-to-hyperintense lesion on T1-weighted images with contrast enhancement [16]. Cavernous angioma appears as a mixed image of hyper and hypointense signals on T1 and T2-weighted sequences, surrounded by a ring of hypointense signals on T2-weighted sequences [9]. The absence of some of these findings made this hypothesis less likely. Metastases also reveal characteristics of highly hemorrhagic lesions; however, the absence of a primary tumor uptake on PET/CT allowed the exclusion of this diagnosis.

The intracanal location of the lesion, as evidenced by MRI, highlights the complexity of the surgical approach that entails careful excision to preserve adjacent neural structures. In this case, it was necessary to section a spinal root, given the adherent and bleeding nature of the lesion, which had to be fragmented.

Histopathological diagnosis is often challenging, given the morphological similarity to other vascular lesions. Immunohistochemistry plays a fundamental role in diagnostic confirmation, being essential in the differentiation of angiosarcoma from other vascular lesions, as observed in this case. Positivity for CD31, CD34, and ERG indicated two diagnostic possibilities, namely epithelioid hemangioendothelioma or angiosarcoma. The negativity for the WWTR1-CAMTA1 fusion gene, which was positive in more than 90% of the cases of epithelioid hemangioendothelioma, confirmed the hypothesis of angiosarcoma [14].

Due to the non-metastatic stage of the disease, the therapeutic approach was multidisciplinary, involving surgery, adjuvant RT, and rehabilitation. Given the difficulty in resecting these lesions, performing a microscopically margin-negative resection surgery was impossible, theoretically giving it a worse prognosis. Adjuvant RT may have played a vital role in the increased survival and greater local control of the disease [8].

After the intervention, the patient showed a favorable response, with improved neurological function and pain control. The addition of motor rehabilitation allowed for a marked functional recovery. Evolution over time highlights the importance of regular follow-up and evaluation by imaging tests. The stability of the lesion on MRI and the improvement of symptoms indicate a positive response to the instituted treatment and an apparent success. However, sustained vigilance is crucial due to the aggressive nature of angiosarcoma and the risk of recurrence.

## Conclusions

The presented clinical case illustrates a rare example of extramedullary intradural primary spinal angiosarcoma, highlighting the challenges associated with the diagnosis of this tumor. The multidisciplinary approach, including surgery, adjuvant RT, and rehabilitation, has been shown to be effective in improving symptoms and stabilizing the disease. The rarity of angiosarcoma in this location highlights the need for a greater understanding of this entity, both in terms of its clinical presentation and response to different therapeutic modalities. Long-term surveillance is vital in detecting early recurrence signs and adjusting the treatment plan. Despite the generally poor prognosis associated with angiosarcoma, the favorable treatment response and successful symptom management observed in this patient underscore the critical importance of a multidisciplinary approach. Future research is needed to improve the therapeutic strategies available for this malignancy.
